# Beauty at a glance: The feeling of beauty and the amplitude of pleasure are independent of stimulus duration

**DOI:** 10.1167/17.14.9

**Published:** 2017-12-11

**Authors:** Aenne A. Brielmann, Lauren Vale, Denis G. Pelli

**Affiliations:** aenne.brielmann@nyu.edu https://aenneb.github.io/; lnv219@nyu.edu; denis.pelli@nyu.edu http://psych.nyu.edu/pelli/; Department of Psychology, New York University, New York, USA; Department of Psychology, New York University, New York, USA; Department of Psychology and Center for Neural Science, New York University, New York, USA

**Keywords:** *stimulus duration*, *beauty*, *aesthetics*, *pleasure*

## Abstract

Over time, how does beauty develop and decay? Common sense suggests that beauty is intensely felt only after prolonged experience of the object. Here, we present one of various stimuli for a variable duration (1–30 s), measure the observers' pleasure over time, and, finally, ask whether they felt beauty. On each trial, participants (*N* = 21) either see an image that they had chosen as “movingly beautiful,” see an image with prerated valence, or suck a candy. During the stimulus and a further 60 s, participants rate pleasure continuously using a custom touchscreen web app, EmotionTracker.com. After each trial, participants judge whether they felt beauty. Across all stimulus kinds, durations, and beauty responses, the dynamic pleasure rating has a stereotypical time course that is well fit by a one-parameter model with a brief exponential onset (roughly 2.5 s), a sustained plateau during stimulus presentation, and a long exponential decay (roughly 70 s). Across conditions, only the plateau amplitude varies. Beauty and pleasure amplitude are nearly independent of stimulus duration. The final beauty rating is positively correlated with pleasure amplitude (*r* = 0.60), and nearly independent of duration (*r* = 0.10). Beauty's independence from duration is unlike Bentham's 18th-century notion of value (utility), which he supposed to depend on the product of pleasure amplitude and duration. Participants report having felt pleasure as strongly after a mere 1 s stimulus as after longer durations, up to 30 s. Thus, we find that amplitude of pleasure is independent of stimulus duration.

## Introduction

How long must one look at an image to experience beauty? In museums, the average looking time for a painting is 27–38 s (Brieber, Nadal, Leder, & Rosenberg, 2014; Smith & Smith, 2001). Yet, most studies in experimental aesthetics limit viewing time to only a few seconds or less (e.g., Forster, Gerger, & Leder, 2015; Forster, Leder, & Ansorge, 2016; Gerger, Leder, Tinio, & Schacht, 2011; Guo, Liu, & Roebuck, 2011; Jakesch, Leder, & Forster, 2013; Kuraguchi & Ashida, 2015). Might such a limited time for sensing the object limit the beauty felt, or is beauty immediate?

### Aesthetic judgments of beauty

People need only a glimpse to make some judgments. A less-than-100-ms presentation suffices to judge the basic-level content of a scene or the attractiveness of a face (Greene & Oliva, 2009; Willis & Todorov, 2006). However, given the social and evolutionary importance of basic categories and facial attractiveness, these tasks could be special cases. Facial attractiveness is usually studied apart from other forms of aesthetic judgment, like beauty.

The mechanisms underlying judgments about the beauty or other aesthetic properties have only recently been subjected to systematic empirical investigation (for reviews see Leder, 2013; Pelowski, Markey, Lauring, & Leder, 2016; Starr, 2013). Some models for the processes underlying aesthetic experiences and judgments have been proposed (Pelowski et al., 2016). One salient structural feature of all these models is that aesthetic judgments (e.g., about beauty) always necessitate a post-sensory, more deliberate stage of cognitive processing. This subsequent processing stage implies that the processing time for aesthetic evaluation should be longer than for a simple judgment of a basic perceptual property such as size or symmetry. In one experiment in which participants made precisely these judgments, an affirmative aesthetic rating (“yes, beautiful”) took about 120 ms longer than basic perceptual judgments (Jacobsen, Schubotz, Höfel, & Cramon, 2006). This raises the question of whether longer processing is only required for (highly) positive aesthetic evaluations, such as: “This is beautiful.”

Philosophers have suggested, and it is widely believed, that beauty is different from other kinds of pleasure (Kant, 1790/2000; Santayana, 1896). This has recently been confirmed experimentally. A brain imaging study showed that most highly moving paintings, but not less-moving ones, co-activated the default-mode network as well as perceptual processing networks (Vessel, Starr, & Rubin, 2012). Brielmann and Pelli (2017) found that using a secondary task to reduce cognitive capacity diminished the pleasure and beauty felt only from beautiful and not from nonbeautiful stimuli. These results suggest that fully experiencing beautiful stimuli requires more cognitive (and possibly more time-consuming) processing than nonbeautiful stimuli. Thus, short presentation durations might prematurely terminate this process and prevent people from achieving a full beauty experience.

### Stimulus duration in experimental aesthetics

Stimulus duration is often a crucial parameter of the experimental design in experimental aesthetics, but the influence of stimulus durations beyond 1 s have not received much attention. Indeed, one study recently found that pleasantness and familiarity ratings for pictures of the International Affective Picture System (IAPS) and paintings are higher when images are presented for 5 s rather than 1 or 25 s (Marin & Leder, 2016). Yet, this effect was small, and, in an additional experiment, did not extend to ratings of liking. Thus, as these authors note, the effect of presentation duration on aesthetic experiences needs further investigation.

Experimenters have used a wide range of stimulus durations in studies looking at aesthetic responses. Stimuli are presented for about 100 ms in forced-choice paradigms involving faces (Guo et al., 2011; Kuraguchi & Ashida, 2015), but up to 5 min when showing art to children (Schabmann et al., 2015). Presumably for practical reasons, controlled stimulus durations tend to be brief, typically less than 10 s. If beauty takes time, then short stimulus durations might preclude the full experience.

In other studies, stimulus duration is controlled, not by the experimenter, but by the participants themselves. That is, participants experienced the stimulus as long as they wished before giving their response, either in a lab (e.g., Augustin & Leder, 2006; Locher, Krupinski, & Schaefer, 2015; Millis, 2001) or while being observed in an art gallery (Kontson et al., 2015; Pelowski, 2015). In those studies, stimulus duration was neither controlled nor a variable of interest. In other studies, self-determined stimulus duration (viewing time) is the dependent variable. Viewing time has been used to measure preference in infants (Bayet et al., 2015; Langlois, Ritter, Roggman, & Vaughn, 1991; Liu et al., 2015; Ramsey, Langlois, Hoss, Rubenstein, & Griffin, 2004; Slater et al., 1998) and predicts adult preference for abstract colored shapes (Holmes & Zanker, 2012, 2013). Viewing time has also been used to measure the extent to which a stimulus is desired (“wanting”) in key-press tasks that allow the participant to prolong viewing by repeatedly pressing a key (Aharon et al., 2001; Dai, Brendl, & Ariely, 2010; Parsons, Young, Kumari, Stein, & Kringelbach, 2011; Sprengelmeyer, Lewis, Hahn, & Perrett, 2013; Wang et al., 2015). The number of key presses then indicates how much participants “want” the stimulus—that is, how much effort they are willing to exert prolonging the experience. To an economist, finding that people will work to prolong pleasant stimuli implies that value increases with duration. This accords with Bentham's (1789/2007) suggestion that value is fundamentally the product of pleasure and duration.

The idea that things are liked more when experienced for longer is also present within the framework of processing fluency theory (Reber, Winkielman, & Schwarz, 1998). These researchers do not claim a direct effect of stimulus duration on liking. Rather, they suppose that longer stimulus presentation increases the ease of processing. To assess the influence of processing fluency on aesthetic judgment, they test various stimulus durations in the range of 100–1000 ms (Forster et al., 2015; Forster et al., 2016; Gerger et al., 2011; Jakesch et al., 2013).

Such studies of the link between fluency of processing and aesthetic liking either show no effect or a positive effect of increasing stimulus duration. Forster and colleagues (2015) disentangled stimulus duration and ease of processing of the stimulus image by independently manipulating stimulus duration (100–400 ms) and the addition of visual noise. They found that liking increased with stimulus duration (100–400 ms). In a later study, Forster and colleagues (2016) found that the rating of the positivity of the stimulus increased with duration (100–400 ms), but that rating of the observer's feelings in response to the stimulus did not change. Jakesch and colleagues (2013) found no net increase of liking for durations of 100–500 ms. Studies with physiological measures show similarly mixed results. In a facial electromyography (fEMG) study, Gerger and colleagues (2011) found that stimulus duration mattered only for abstract patterns, not for faces. Activation of the smile muscle Zygomaticus Major grew with duration of presentation (47 vs. 400 ms) of abstract patterns, indicating a more positive affective response. No duration effect was observed for face stimuli. Combining self-reports, fEMG, and skin conductance responses, Forster and colleagues (2016) found a decrease in activation of the frown muscle Corrugator Supercilii with stimulus duration (100 to 400 ms), but no change in Zygomaticus Major activity.

Apart from these visual studies, a recent music study suggests that liking may be insensitive to music duration. Belfi, Rowland, Vessel, Starr, and Poeppel (2016) found that people can tell whether they will like a 10-s excerpt after hearing just 750 ms of it.

### Current study

In sum, across self-reports and physiological measures, there is some evidence for small positive effects of increasing stimulus duration on aesthetic liking. This effect is often attributed to increased fluency of processing (but see Jakesch et al., 2013). These results are restricted to a set of artificial stimuli, as opposed to, for example, faces (Gerger et al., 2011). The stimulus durations studied so far are less than 1 s. Thus, the question remains open whether stimulus duration would also affect aesthetic judgments in a less artificial setting—that is, when stimuli are more complex, potentially familiar to participants, and experienced for more than 1 s.

Using a continuous measure to track self-reported pleasure through the entire course of the trial, we examined how the stimulus duration affects the experience of beauty. We not only measured the end point of the aesthetic value judgment (here: beauty) but with our custom touchscreen web app EmotionTracker.com, we measured the dynamic unfolding of pleasure over time both during and well beyond the stimulus presentation. Here, we investigate whether stimulus duration affects the feeling of beauty and the time course of pleasure. Specifically, we ask whether the growth with stimulus duration (50–500 ms) observed in some past studies extrapolates to longer durations (1–30 s). Heeding the trope that high beauty requires prolonged contemplation, we also test whether longer durations specifically increase pleasure only of experiences rated high in beauty. In addition, as positive duration effects were not observed for all stimuli investigated in past studies, we ask whether stimulus duration has different effects on different kinds of stimuli.

## Methods

### Participants

Twenty-one naive observers were recruited from the immediate environment of New York University. All were adults and gave written informed consent according to the University Committee on Activities Involving Human Subjects. Participants received either $10 or course credit as compensation.

### Stimuli

Participants saw four categories of images and sucked a piece of candy. Images were presented on a 21.5-in. iMac display covering the entire width of the screen from a distance of approximately 1 m. A range of images was provided to increase the likelihood that participants would experience beauty, as well as mild pleasure and neutral feelings. First, all observers were asked to provide four to six images that were “movingly beautiful” to them (see also Vessel et al., 2012; Vale, Gerger, Leder, & Pelli, 2017). We will refer to these images as *self-selected images*. Self-selected beautiful images were chosen because they reliably elicit high beauty and pleasure (Brielmann & Pelli, 2017). Participants' selections are highly diverse and span diverse motifs, ranging from landscape photographs to art to one participant picking a photograph of her fiancé. Second, we selected six images from the IAPS (Lang, Bradley, & Cuthbert, 2008) that had extremely positive valence (7–8 on a scale from 1–8) and elevated arousal ratings (5–6 on a scale from 1–8 [high-valence IAPS]; picture numbers 1710, 5600, 5621, 5833, 7330, 7508). We excluded erotic images. Third, we chose the same number of images from the IAPS database that had slightly positive valence (5–7) and medium arousal (3–4) ratings to provide a moderately pleasurable stimulus within the same modality as the beautiful stimuli (mid-valence IAPS; picture numbers 1947, 7281, 7545, 7160, 5711, 7340). Fourth, images of IKEA furniture against a white background were presented as neutral images (www.ikea.com). Fifth, we let participants suck various flavors of hard candy (Hershey's Jolly Rancher) to provide a pleasurable experience in another sense modality. Some philosophers have claimed that sensuous pleasures cannot be beautiful (e.g., Kant, 1790/2000, but see Brielmann & Pelli, 2017), so, for diversity, we wanted to include a sensuous pleasure. We presumed that sucking candy would lead participants to experience great pleasure without necessarily evoking the experience of beauty.

### Procedure

Each trial presents an image or candy for 1, 6, 15, or 30 s, followed by a blank screen, and lasted 90 s in total. Stimulus presentation for the candy was ended by asking the participant to spit the candy into an empty cup and to rinse his or her mouth with water. Twenty trials were presented in random order. Each participant experienced each stimulus kind four times, once per duration. For each duration, we randomized the flavor of the candy tasted and the individual image shown. Participants were asked to continuously rate their pleasure from the image or candy for the entire trial. Continuous pleasure ratings were made by dynamically adjusting the spread of the fingertips of their index and middle finger of their dominant hand on an iPad running our custom web app EmotionTracker.com. Comfortably maintainable maximum finger spread indicated highest pleasure, and minimal finger spread indicated minimal pleasure. Both extreme finger spreads were recorded at the beginning of the participants' first trial and served to linearly map finger spread sampled at 1 Hz to numeric ratings on a 1–10 scale.

Pretests documented the accuracy of EmotionTracker.com ratings. Those participants were asked to track with finger spread a visually presented sequence of pseudorandom numbers between 1 and 9 (*n* = 3; four trials each) or the rounded numbers previously obtained from their actual ratings obtained while viewing images (*n* = 1; five trials). Pseudorandom numbers were independent samples from a uniform distribution of the integers 1–9. A new number was presented every 3 s. Root mean square error (RMSE) was on average 1.0 for tracking of actual ratings and 1.5 for tracking of random numbers. Results are shown in the Supplementary Material. The results of this pilot test show that people using the emotion tracker app can accurately control the spread of their fingers to continuously track a varying stimulus parameter. This makes it reasonable to suppose that they can similarly track their internal pleasure.

After continuously rating their pleasure, at the end of the trial, participants were asked “During this trial, did you get the feeling of beauty from the object?” They answered on a 4-point scale: *Definitely not* (0), *Perhaps not* (1), *Perhaps yes* (2), and *Definitely yes* (3). The numbers indicate our encoding, and were not displayed to the participants.

Pleasure and beauty ratings in our study were thus collected through two different kinds of response scale and modality. Pleasure was rated on a continuous analog scale of finger spread, during the stimulus and beyond, whereas beauty was rated on a discrete verbal scale, well after the stimulus. These very different response modes were chosen to minimize artefactual correlation between beauty and pleasure ratings due to observers inadvertently failing to distinguish them, and just repeating a rating response without regard to the question. Previous work in our lab has shown that the amplitudes of such pleasure and beauty ratings are positively correlated with each other: moderately on a trial-by-trial basis and strongly when looking at stimulus averages (Brielmann & Pelli, 2017).

### Analyses

**Figure 1:**
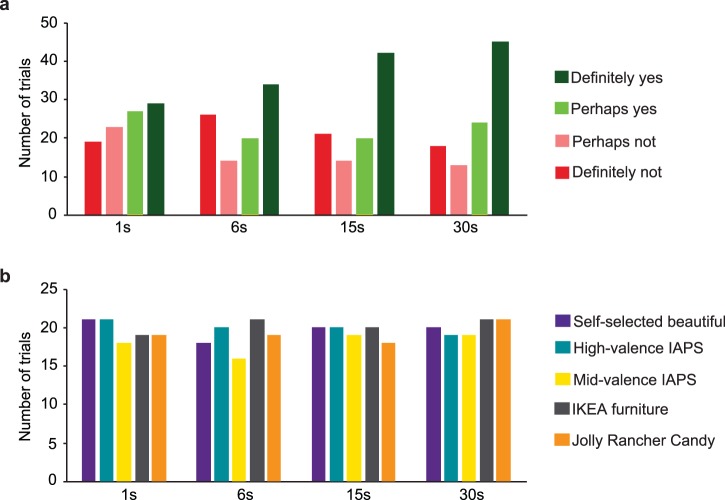
Number of trials for each duration sorted by beauty judgment (a) and stimulus type (b). (a) Colors indicate final beauty judgments: dark red = Definitely not; light red = Perhaps not; light green = Perhaps yes; dark green = Definitely yes. The frequency of the Definitely beautiful response increases with duration, but the increase is—especially given that this was a nonplanned test and the low number of data points—far from significant, r(2) = 0.94, p = 0.06, 95% CI [−0.22, 1.00]. (b) Colors indicate stimulus category: violet = self-selected beautiful; turquoise = high-valence IAPS; yellow = mid-valence IAPS; gray = IKEA furniture; orange = Jolly Rancher candy.

Data from 31 trials were lost due to technical problems with the EmotionTracker.com app. Figure 1 shows the exact number of ratings per duration and stimulus category. Thus, data analyses included the remaining 389 trials (*N* = 21). Data processing, model fitting, and bootstrapping were conducted with MATLAB (version 2010a or higher; MathWorks, Inc., Natick, MA, USA) and statistical analyses with R (version 3.2.3).^1^ To assess whether participants' final beauty judgments were influenced by stimulus duration, we used a 4 × 5 (Duration × Stimulus type) analysis of variance (ANOVA). Tukey's honest significant distance (HSD) was used for post hoc multiple comparisons. Cohen's *d* was calculated as a measure of effect size for significant differences. All reported correlations are Pearson's correlations.

## Results

### A simple model of the dynamics of pleasure

Inspection of continuous pleasure ratings led us to suppose a simple mathematical model to summarize our data. The model makes exponential transitions among three stable states: initial, steady-state, and final. The response level is *r*_initial_ until stimulus onset time $\newcommand{\bialpha}{\boldsymbol{\alpha}}\newcommand{\bibeta}{\boldsymbol{\beta}}\newcommand{\bigamma}{\boldsymbol{\gamma}}\newcommand{\bidelta}{\boldsymbol{\delta}}\newcommand{\bivarepsilon}{\boldsymbol{\varepsilon}}\newcommand{\bizeta}{\boldsymbol{\zeta}}\newcommand{\bieta}{\boldsymbol{\eta}}\newcommand{\bitheta}{\boldsymbol{\theta}}\newcommand{\biiota}{\boldsymbol{\iota}}\newcommand{\bikappa}{\boldsymbol{\kappa}}\newcommand{\bilambda}{\boldsymbol{\lambda}}\newcommand{\bimu}{\boldsymbol{\mu}}\newcommand{\binu}{\boldsymbol{\nu}}\newcommand{\bixi}{\boldsymbol{\xi}}\newcommand{\biomicron}{\boldsymbol{\micron}}\newcommand{\bipi}{\boldsymbol{\pi}}\newcommand{\birho}{\boldsymbol{\rho}}\newcommand{\bisigma}{\boldsymbol{\sigma}}\newcommand{\bitau}{\boldsymbol{\tau}}\newcommand{\biupsilon}{\boldsymbol{\upsilon}}\newcommand{\biphi}{\boldsymbol{\phi}}\newcommand{\bichi}{\boldsymbol{\chi}}\newcommand{\bipsi}{\boldsymbol{\psi}}\newcommand{\biomega}{\boldsymbol{\omega}}{t_{{\rm{on}}}}$, then exponentially approaches the steady-state response *r*_steady_ until stimulus offset time $\newcommand{\bialpha}{\boldsymbol{\alpha}}\newcommand{\bibeta}{\boldsymbol{\beta}}\newcommand{\bigamma}{\boldsymbol{\gamma}}\newcommand{\bidelta}{\boldsymbol{\delta}}\newcommand{\bivarepsilon}{\boldsymbol{\varepsilon}}\newcommand{\bizeta}{\boldsymbol{\zeta}}\newcommand{\bieta}{\boldsymbol{\eta}}\newcommand{\bitheta}{\boldsymbol{\theta}}\newcommand{\biiota}{\boldsymbol{\iota}}\newcommand{\bikappa}{\boldsymbol{\kappa}}\newcommand{\bilambda}{\boldsymbol{\lambda}}\newcommand{\bimu}{\boldsymbol{\mu}}\newcommand{\binu}{\boldsymbol{\nu}}\newcommand{\bixi}{\boldsymbol{\xi}}\newcommand{\biomicron}{\boldsymbol{\micron}}\newcommand{\bipi}{\boldsymbol{\pi}}\newcommand{\birho}{\boldsymbol{\rho}}\newcommand{\bisigma}{\boldsymbol{\sigma}}\newcommand{\bitau}{\boldsymbol{\tau}}\newcommand{\biupsilon}{\boldsymbol{\upsilon}}\newcommand{\biphi}{\boldsymbol{\phi}}\newcommand{\bichi}{\boldsymbol{\chi}}\newcommand{\bipsi}{\boldsymbol{\psi}}\newcommand{\biomega}{\boldsymbol{\omega}}{t_{{\rm{off}}}}$, and then decays exponentially towards the final level *r*_final_. Two time constants τ_short_ and τ_long_ and a weight *w*_short_ set the speed of the transitions. The initial transition is exponential. The final transition is the sum of two exponentials: a fast one with the same time constant as the initial increase τ_short_, and a slower one with the time constant τ_long_. The relative weight of the fast and slow components is set by *w*_short_. Equations 1 through 3 define the model:

\newcommand{\bialpha}{\boldsymbol{\alpha}}\newcommand{\bibeta}{\boldsymbol{\beta}}\newcommand{\bigamma}{\boldsymbol{\gamma}}\newcommand{\bidelta}{\boldsymbol{\delta}}\newcommand{\bivarepsilon}{\boldsymbol{\varepsilon}}\newcommand{\bizeta}{\boldsymbol{\zeta}}\newcommand{\bieta}{\boldsymbol{\eta}}\newcommand{\bitheta}{\boldsymbol{\theta}}\newcommand{\biiota}{\boldsymbol{\iota}}\newcommand{\bikappa}{\boldsymbol{\kappa}}\newcommand{\bilambda}{\boldsymbol{\lambda}}\newcommand{\bimu}{\boldsymbol{\mu}}\newcommand{\binu}{\boldsymbol{\nu}}\newcommand{\bixi}{\boldsymbol{\xi}}\newcommand{\biomicron}{\boldsymbol{\micron}}\newcommand{\bipi}{\boldsymbol{\pi}}\newcommand{\birho}{\boldsymbol{\rho}}\newcommand{\bisigma}{\boldsymbol{\sigma}}\newcommand{\bitau}{\boldsymbol{\tau}}\newcommand{\biupsilon}{\boldsymbol{\upsilon}}\newcommand{\biphi}{\boldsymbol{\phi}}\newcommand{\bichi}{\boldsymbol{\chi}}\newcommand{\bipsi}{\boldsymbol{\psi}}\newcommand{\biomega}{\boldsymbol{\omega}}\begin{equation}\tag{1}\hat {R} = {\alpha _{{\rm{on}}}}\left( t \right){r_{{\rm{initial}}}} + \left( {1 - {\alpha _{{\rm{on}}}}(t)} \right)\left[ {{\alpha _{{\rm{off}}}}\left( t \right){r_{{\rm{steady}}}} + \left( {1 - {\alpha _{{\rm{off}}}}(t)} \right){r_{{\rm{final}}}}} \right]\end{equation}

\newcommand{\bialpha}{\boldsymbol{\alpha}}\newcommand{\bibeta}{\boldsymbol{\beta}}\newcommand{\bigamma}{\boldsymbol{\gamma}}\newcommand{\bidelta}{\boldsymbol{\delta}}\newcommand{\bivarepsilon}{\boldsymbol{\varepsilon}}\newcommand{\bizeta}{\boldsymbol{\zeta}}\newcommand{\bieta}{\boldsymbol{\eta}}\newcommand{\bitheta}{\boldsymbol{\theta}}\newcommand{\biiota}{\boldsymbol{\iota}}\newcommand{\bikappa}{\boldsymbol{\kappa}}\newcommand{\bilambda}{\boldsymbol{\lambda}}\newcommand{\bimu}{\boldsymbol{\mu}}\newcommand{\binu}{\boldsymbol{\nu}}\newcommand{\bixi}{\boldsymbol{\xi}}\newcommand{\biomicron}{\boldsymbol{\micron}}\newcommand{\bipi}{\boldsymbol{\pi}}\newcommand{\birho}{\boldsymbol{\rho}}\newcommand{\bisigma}{\boldsymbol{\sigma}}\newcommand{\bitau}{\boldsymbol{\tau}}\newcommand{\biupsilon}{\boldsymbol{\upsilon}}\newcommand{\biphi}{\boldsymbol{\phi}}\newcommand{\bichi}{\boldsymbol{\chi}}\newcommand{\bipsi}{\boldsymbol{\psi}}\newcommand{\biomega}{\boldsymbol{\omega}}\begin{equation}\tag{2}{\alpha _{{\rm{on}}}}\left( t \right) = {\rm{exp}}{{ - \lfloor t - {t_{{\rm{on}}}}\rfloor } \over {{\tau _{{\rm{short}}}}}}{\rm .}\end{equation}

\newcommand{\bialpha}{\boldsymbol{\alpha}}\newcommand{\bibeta}{\boldsymbol{\beta}}\newcommand{\bigamma}{\boldsymbol{\gamma}}\newcommand{\bidelta}{\boldsymbol{\delta}}\newcommand{\bivarepsilon}{\boldsymbol{\varepsilon}}\newcommand{\bizeta}{\boldsymbol{\zeta}}\newcommand{\bieta}{\boldsymbol{\eta}}\newcommand{\bitheta}{\boldsymbol{\theta}}\newcommand{\biiota}{\boldsymbol{\iota}}\newcommand{\bikappa}{\boldsymbol{\kappa}}\newcommand{\bilambda}{\boldsymbol{\lambda}}\newcommand{\bimu}{\boldsymbol{\mu}}\newcommand{\binu}{\boldsymbol{\nu}}\newcommand{\bixi}{\boldsymbol{\xi}}\newcommand{\biomicron}{\boldsymbol{\micron}}\newcommand{\bipi}{\boldsymbol{\pi}}\newcommand{\birho}{\boldsymbol{\rho}}\newcommand{\bisigma}{\boldsymbol{\sigma}}\newcommand{\bitau}{\boldsymbol{\tau}}\newcommand{\biupsilon}{\boldsymbol{\upsilon}}\newcommand{\biphi}{\boldsymbol{\phi}}\newcommand{\bichi}{\boldsymbol{\chi}}\newcommand{\bipsi}{\boldsymbol{\psi}}\newcommand{\biomega}{\boldsymbol{\omega}}\begin{equation}\tag{3}{\alpha _{{\rm{off}}}}\left( t \right) = {w_{{\rm{short}}}}\exp {{ - \lfloor t - {t_{{\rm{off}}}}\rfloor } \over {{\tau _{{\rm{short}}}}}} + (1 - {w_{{\rm{short}}}})\exp {{ - \lfloor t - {t_{{\rm{off}}}}\rfloor } \over {{\tau _{{\rm{long}}}}}}\end{equation}

where ⌊*x*⌋ = max(0, *x*) is the “floor” function, and $\newcommand{\bialpha}{\boldsymbol{\alpha}}\newcommand{\bibeta}{\boldsymbol{\beta}}\newcommand{\bigamma}{\boldsymbol{\gamma}}\newcommand{\bidelta}{\boldsymbol{\delta}}\newcommand{\bivarepsilon}{\boldsymbol{\varepsilon}}\newcommand{\bizeta}{\boldsymbol{\zeta}}\newcommand{\bieta}{\boldsymbol{\eta}}\newcommand{\bitheta}{\boldsymbol{\theta}}\newcommand{\biiota}{\boldsymbol{\iota}}\newcommand{\bikappa}{\boldsymbol{\kappa}}\newcommand{\bilambda}{\boldsymbol{\lambda}}\newcommand{\bimu}{\boldsymbol{\mu}}\newcommand{\binu}{\boldsymbol{\nu}}\newcommand{\bixi}{\boldsymbol{\xi}}\newcommand{\biomicron}{\boldsymbol{\micron}}\newcommand{\bipi}{\boldsymbol{\pi}}\newcommand{\birho}{\boldsymbol{\rho}}\newcommand{\bisigma}{\boldsymbol{\sigma}}\newcommand{\bitau}{\boldsymbol{\tau}}\newcommand{\biupsilon}{\boldsymbol{\upsilon}}\newcommand{\biphi}{\boldsymbol{\phi}}\newcommand{\bichi}{\boldsymbol{\chi}}\newcommand{\bipsi}{\boldsymbol{\psi}}\newcommand{\biomega}{\boldsymbol{\omega}}{t_{{\rm{on}}}}$ and $\newcommand{\bialpha}{\boldsymbol{\alpha}}\newcommand{\bibeta}{\boldsymbol{\beta}}\newcommand{\bigamma}{\boldsymbol{\gamma}}\newcommand{\bidelta}{\boldsymbol{\delta}}\newcommand{\bivarepsilon}{\boldsymbol{\varepsilon}}\newcommand{\bizeta}{\boldsymbol{\zeta}}\newcommand{\bieta}{\boldsymbol{\eta}}\newcommand{\bitheta}{\boldsymbol{\theta}}\newcommand{\biiota}{\boldsymbol{\iota}}\newcommand{\bikappa}{\boldsymbol{\kappa}}\newcommand{\bilambda}{\boldsymbol{\lambda}}\newcommand{\bimu}{\boldsymbol{\mu}}\newcommand{\binu}{\boldsymbol{\nu}}\newcommand{\bixi}{\boldsymbol{\xi}}\newcommand{\biomicron}{\boldsymbol{\micron}}\newcommand{\bipi}{\boldsymbol{\pi}}\newcommand{\birho}{\boldsymbol{\rho}}\newcommand{\bisigma}{\boldsymbol{\sigma}}\newcommand{\bitau}{\boldsymbol{\tau}}\newcommand{\biupsilon}{\boldsymbol{\upsilon}}\newcommand{\biphi}{\boldsymbol{\phi}}\newcommand{\bichi}{\boldsymbol{\chi}}\newcommand{\bipsi}{\boldsymbol{\psi}}\newcommand{\biomega}{\boldsymbol{\omega}}{t_{{\rm{off}}}}$ are stimulus onset and offset. In fitting the model, we allow only the parameter $\newcommand{\bialpha}{\boldsymbol{\alpha}}\newcommand{\bibeta}{\boldsymbol{\beta}}\newcommand{\bigamma}{\boldsymbol{\gamma}}\newcommand{\bidelta}{\boldsymbol{\delta}}\newcommand{\bivarepsilon}{\boldsymbol{\varepsilon}}\newcommand{\bizeta}{\boldsymbol{\zeta}}\newcommand{\bieta}{\boldsymbol{\eta}}\newcommand{\bitheta}{\boldsymbol{\theta}}\newcommand{\biiota}{\boldsymbol{\iota}}\newcommand{\bikappa}{\boldsymbol{\kappa}}\newcommand{\bilambda}{\boldsymbol{\lambda}}\newcommand{\bimu}{\boldsymbol{\mu}}\newcommand{\binu}{\boldsymbol{\nu}}\newcommand{\bixi}{\boldsymbol{\xi}}\newcommand{\biomicron}{\boldsymbol{\micron}}\newcommand{\bipi}{\boldsymbol{\pi}}\newcommand{\birho}{\boldsymbol{\rho}}\newcommand{\bisigma}{\boldsymbol{\sigma}}\newcommand{\bitau}{\boldsymbol{\tau}}\newcommand{\biupsilon}{\boldsymbol{\upsilon}}\newcommand{\biphi}{\boldsymbol{\phi}}\newcommand{\bichi}{\boldsymbol{\chi}}\newcommand{\bipsi}{\boldsymbol{\psi}}\newcommand{\biomega}{\boldsymbol{\omega}}{r_{{\rm{steady}}}}$ to vary across conditions. The five *general* parameters *r*_initial_, *r*_final_, *τ*_short_, *τ*_long_, and $\newcommand{\bialpha}{\boldsymbol{\alpha}}\newcommand{\bibeta}{\boldsymbol{\beta}}\newcommand{\bigamma}{\boldsymbol{\gamma}}\newcommand{\bidelta}{\boldsymbol{\delta}}\newcommand{\bivarepsilon}{\boldsymbol{\varepsilon}}\newcommand{\bizeta}{\boldsymbol{\zeta}}\newcommand{\bieta}{\boldsymbol{\eta}}\newcommand{\bitheta}{\boldsymbol{\theta}}\newcommand{\biiota}{\boldsymbol{\iota}}\newcommand{\bikappa}{\boldsymbol{\kappa}}\newcommand{\bilambda}{\boldsymbol{\lambda}}\newcommand{\bimu}{\boldsymbol{\mu}}\newcommand{\binu}{\boldsymbol{\nu}}\newcommand{\bixi}{\boldsymbol{\xi}}\newcommand{\biomicron}{\boldsymbol{\micron}}\newcommand{\bipi}{\boldsymbol{\pi}}\newcommand{\birho}{\boldsymbol{\rho}}\newcommand{\bisigma}{\boldsymbol{\sigma}}\newcommand{\bitau}{\boldsymbol{\tau}}\newcommand{\biupsilon}{\boldsymbol{\upsilon}}\newcommand{\biphi}{\boldsymbol{\phi}}\newcommand{\bichi}{\boldsymbol{\chi}}\newcommand{\bipsi}{\boldsymbol{\psi}}\newcommand{\biomega}{\boldsymbol{\omega}}{w_{{\rm{short}}}}$ are each allowed one value across all conditions.

We used MATLAB to fit this model to pleasure over time averaged across trials either for each duration and final beauty judgment or for each duration and stimulus type. Initial values for the parameters were: *r*_initial_ = 1, *r*_steady_ = 5, *r*_final_ = 1, τ_short_ = 1, τ_long_ = 60 s, and *w*_short_ = 0.5. Each fit's RMSE is reported in Table 1. Plotted residuals of model fits over time are in the Supplementary Material. In fitting the averages by beauty judgment, the general parameter values were *r*_initial_ = 1.10, *r*_final_ = 1.37, τ_short_ = 2.20, τ_long_ = 59.5, and *w*_short_ = 0.033. Similar values were obtained for fitting the averages by stimulus category: *r*_initial_ = 1.63, *r*_final_ = 1.00, τ_short_ = 2.77, τ_long_ = 79.9, and *w*_short_ = 0.119. We also fit the model to single-trial data, which are noisier than average time courses, and still got a decent fit (RMSE = 1.25) with parameter values of *r*_initial_ = 1.70, *r*_final_ = 0.00, *τ*_short_ = 4.26, *τ*_long_ = 135.8, and *w*_short_ = 0.205.

We then froze all parameters but *r*_steady_, and solved this now one-parameter model analytically (Equation 4) for minimum RMSE $\newcommand{\bialpha}{\boldsymbol{\alpha}}\newcommand{\bibeta}{\boldsymbol{\beta}}\newcommand{\bigamma}{\boldsymbol{\gamma}}\newcommand{\bidelta}{\boldsymbol{\delta}}\newcommand{\bivarepsilon}{\boldsymbol{\varepsilon}}\newcommand{\bizeta}{\boldsymbol{\zeta}}\newcommand{\bieta}{\boldsymbol{\eta}}\newcommand{\bitheta}{\boldsymbol{\theta}}\newcommand{\biiota}{\boldsymbol{\iota}}\newcommand{\bikappa}{\boldsymbol{\kappa}}\newcommand{\bilambda}{\boldsymbol{\lambda}}\newcommand{\bimu}{\boldsymbol{\mu}}\newcommand{\binu}{\boldsymbol{\nu}}\newcommand{\bixi}{\boldsymbol{\xi}}\newcommand{\biomicron}{\boldsymbol{\micron}}\newcommand{\bipi}{\boldsymbol{\pi}}\newcommand{\birho}{\boldsymbol{\rho}}\newcommand{\bisigma}{\boldsymbol{\sigma}}\newcommand{\bitau}{\boldsymbol{\tau}}\newcommand{\biupsilon}{\boldsymbol{\upsilon}}\newcommand{\biphi}{\boldsymbol{\phi}}\newcommand{\bichi}{\boldsymbol{\chi}}\newcommand{\bipsi}{\boldsymbol{\psi}}\newcommand{\biomega}{\boldsymbol{\omega}}\hat {R} (t)$ − *R*(*t*). For each trial response *R*(*t*), the best RMSE fit $\newcommand{\bialpha}{\boldsymbol{\alpha}}\newcommand{\bibeta}{\boldsymbol{\beta}}\newcommand{\bigamma}{\boldsymbol{\gamma}}\newcommand{\bidelta}{\boldsymbol{\delta}}\newcommand{\bivarepsilon}{\boldsymbol{\varepsilon}}\newcommand{\bizeta}{\boldsymbol{\zeta}}\newcommand{\bieta}{\boldsymbol{\eta}}\newcommand{\bitheta}{\boldsymbol{\theta}}\newcommand{\biiota}{\boldsymbol{\iota}}\newcommand{\bikappa}{\boldsymbol{\kappa}}\newcommand{\bilambda}{\boldsymbol{\lambda}}\newcommand{\bimu}{\boldsymbol{\mu}}\newcommand{\binu}{\boldsymbol{\nu}}\newcommand{\bixi}{\boldsymbol{\xi}}\newcommand{\biomicron}{\boldsymbol{\micron}}\newcommand{\bipi}{\boldsymbol{\pi}}\newcommand{\birho}{\boldsymbol{\rho}}\newcommand{\bisigma}{\boldsymbol{\sigma}}\newcommand{\bitau}{\boldsymbol{\tau}}\newcommand{\biupsilon}{\boldsymbol{\upsilon}}\newcommand{\biphi}{\boldsymbol{\phi}}\newcommand{\bichi}{\boldsymbol{\chi}}\newcommand{\bipsi}{\boldsymbol{\psi}}\newcommand{\biomega}{\boldsymbol{\omega}}\hat {R} \left( t \right)$ by the single-parameter model has steady-state response

\newcommand{\bialpha}{\boldsymbol{\alpha}}\newcommand{\bibeta}{\boldsymbol{\beta}}\newcommand{\bigamma}{\boldsymbol{\gamma}}\newcommand{\bidelta}{\boldsymbol{\delta}}\newcommand{\bivarepsilon}{\boldsymbol{\varepsilon}}\newcommand{\bizeta}{\boldsymbol{\zeta}}\newcommand{\bieta}{\boldsymbol{\eta}}\newcommand{\bitheta}{\boldsymbol{\theta}}\newcommand{\biiota}{\boldsymbol{\iota}}\newcommand{\bikappa}{\boldsymbol{\kappa}}\newcommand{\bilambda}{\boldsymbol{\lambda}}\newcommand{\bimu}{\boldsymbol{\mu}}\newcommand{\binu}{\boldsymbol{\nu}}\newcommand{\bixi}{\boldsymbol{\xi}}\newcommand{\biomicron}{\boldsymbol{\micron}}\newcommand{\bipi}{\boldsymbol{\pi}}\newcommand{\birho}{\boldsymbol{\rho}}\newcommand{\bisigma}{\boldsymbol{\sigma}}\newcommand{\bitau}{\boldsymbol{\tau}}\newcommand{\biupsilon}{\boldsymbol{\upsilon}}\newcommand{\biphi}{\boldsymbol{\phi}}\newcommand{\bichi}{\boldsymbol{\chi}}\newcommand{\bipsi}{\boldsymbol{\psi}}\newcommand{\biomega}{\boldsymbol{\omega}}\begin{equation}\tag{4}{r_{{\rm{steady}}}} = {{{\sum _t}\left( {R\left( t \right) - f\left( t \right)} \right)g\left( t \right)} \over {{\sum _t}{g^2}\left( t \right)}}\end{equation}

where

\newcommand{\bialpha}{\boldsymbol{\alpha}}\newcommand{\bibeta}{\boldsymbol{\beta}}\newcommand{\bigamma}{\boldsymbol{\gamma}}\newcommand{\bidelta}{\boldsymbol{\delta}}\newcommand{\bivarepsilon}{\boldsymbol{\varepsilon}}\newcommand{\bizeta}{\boldsymbol{\zeta}}\newcommand{\bieta}{\boldsymbol{\eta}}\newcommand{\bitheta}{\boldsymbol{\theta}}\newcommand{\biiota}{\boldsymbol{\iota}}\newcommand{\bikappa}{\boldsymbol{\kappa}}\newcommand{\bilambda}{\boldsymbol{\lambda}}\newcommand{\bimu}{\boldsymbol{\mu}}\newcommand{\binu}{\boldsymbol{\nu}}\newcommand{\bixi}{\boldsymbol{\xi}}\newcommand{\biomicron}{\boldsymbol{\micron}}\newcommand{\bipi}{\boldsymbol{\pi}}\newcommand{\birho}{\boldsymbol{\rho}}\newcommand{\bisigma}{\boldsymbol{\sigma}}\newcommand{\bitau}{\boldsymbol{\tau}}\newcommand{\biupsilon}{\boldsymbol{\upsilon}}\newcommand{\biphi}{\boldsymbol{\phi}}\newcommand{\bichi}{\boldsymbol{\chi}}\newcommand{\bipsi}{\boldsymbol{\psi}}\newcommand{\biomega}{\boldsymbol{\omega}}\begin{equation}\tag{5}f\left( t \right) = {\alpha _{{\rm{on}}}}\left( t \right){r_{{\rm{initial}}}} + \left( {1 - {\alpha _{{\rm{on}}}}\left( t \right)} \right)\left( {1 - {\alpha _{{\rm{off}}}}\left( t \right)} \right){r_{{\rm{final}}}}\end{equation}

and

\newcommand{\bialpha}{\boldsymbol{\alpha}}\newcommand{\bibeta}{\boldsymbol{\beta}}\newcommand{\bigamma}{\boldsymbol{\gamma}}\newcommand{\bidelta}{\boldsymbol{\delta}}\newcommand{\bivarepsilon}{\boldsymbol{\varepsilon}}\newcommand{\bizeta}{\boldsymbol{\zeta}}\newcommand{\bieta}{\boldsymbol{\eta}}\newcommand{\bitheta}{\boldsymbol{\theta}}\newcommand{\biiota}{\boldsymbol{\iota}}\newcommand{\bikappa}{\boldsymbol{\kappa}}\newcommand{\bilambda}{\boldsymbol{\lambda}}\newcommand{\bimu}{\boldsymbol{\mu}}\newcommand{\binu}{\boldsymbol{\nu}}\newcommand{\bixi}{\boldsymbol{\xi}}\newcommand{\biomicron}{\boldsymbol{\micron}}\newcommand{\bipi}{\boldsymbol{\pi}}\newcommand{\birho}{\boldsymbol{\rho}}\newcommand{\bisigma}{\boldsymbol{\sigma}}\newcommand{\bitau}{\boldsymbol{\tau}}\newcommand{\biupsilon}{\boldsymbol{\upsilon}}\newcommand{\biphi}{\boldsymbol{\phi}}\newcommand{\bichi}{\boldsymbol{\chi}}\newcommand{\bipsi}{\boldsymbol{\psi}}\newcommand{\biomega}{\boldsymbol{\omega}}\begin{equation}\tag{6}g\left( t \right) = \left( {1 - {\alpha _{{\rm{on}}}}\left( t \right)} \right){\alpha _{{\rm{off}}}}\left( t \right)\end{equation}

In sum, this mathematical model allows us to describe the changing pleasure rating through the entire trial with one number, *r*_steady_. It shows that the rise and fall of pleasure are exponential decays, like many other natural processes, including fluid emptying from a tube and radioactive decay. The same model also fits the data of an independent dataset from our lab (Brielmann & Pelli, 2017).

To confirm that *r*_steady_ pleasure captures an important aspect of the beauty experience, we correlated beauty judgments and *r*_steady_ values for each trial. The correlation between the two measures was considerable for *r*_steady_ values obtained with fixed parameters from average beauty category curves, *r*(387) = 0.60, *p* < 0.001, 95% CI [0.53, 0.66], and average stimulus type curves alike, *r*(387) = 0.59, *p* < 0.001, [0.53, 0.66]. Thus, experienced beauty is moderately correlated with pleasure amplitude.

### Final beauty judgments are only marginally affected by stimulus duration

**Figure 2:**
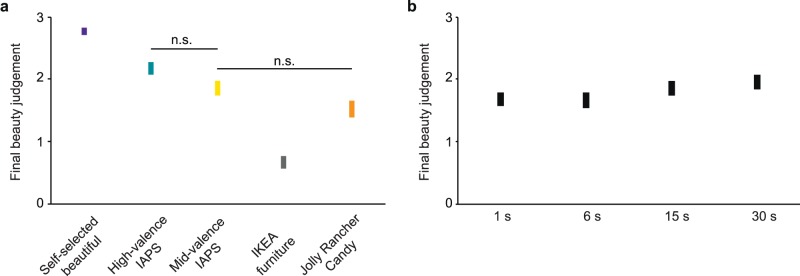
Average final beauty judgments for each stimulus type (a) and duration (b). Boxes represent ± SEM. All differences between stimulus types are significant according to post hoc Tukey honest significance difference tests, all ps < 0.001, if not marked as n.s. (nonsignificant). None of the differences between durations are significant.

Final beauty judgments strongly depended on the stimulus type, *F*(4, 379) = 55.75, *p* < 0.001. Figure 2 illustrates that participants judged the experience of their self-selected images as most beautiful, all *d* ≥ 0.78, followed by both types of IAPS images and the Jolly Rancher candy. Reports of beauty experiences were most rare for IKEA images, all *d* ≤ −0.30. In addition, increasing stimulus duration only weakly increased final beauty judgments, *F*(1, 379) = 7.65, *p* = 0.006, *r*(387) = 0.10, 95% CI [0.00, 0.20], *p* = 0.04. Stimulus type and duration did not interact, *F*(4, 379) = 0.52, *p* = 0.719.

### Presentation duration does not affect pleasure amplitude

**Figure 3:**
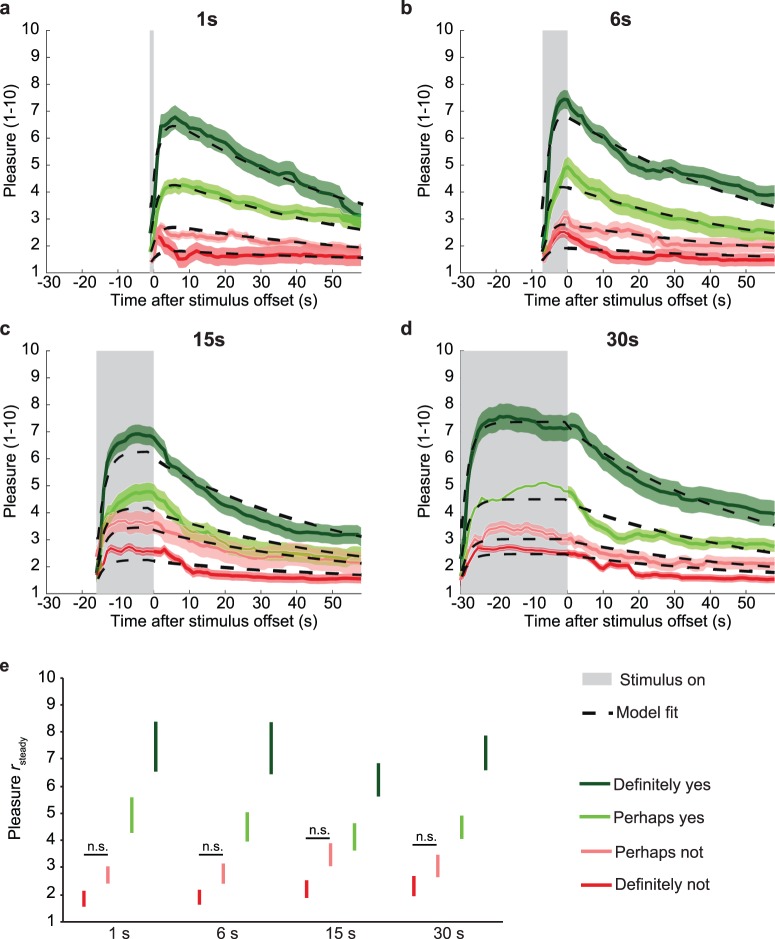
Model fits and r_steady_ for each beauty judgment. (a–d) Time course of pleasure rating (M ± SEM) and corresponding model fits for each beauty judgment for each duration (a: 1 s; b: 6 s; c: 15 s; d: 30 s). Model fits were made with Equations 1 through 3. Solid lines with shaded areas represent the data, dashed black lines model fits. Colors indicate final beauty judgments: dark red = Definitely not; light red = Perhaps not; light green = Perhaps yes; dark green = Definitely yes. The gray shaded area indicates the interval during which the stimulus was present. (e) M ± SEM for r_steady_ for each beauty judgment and duration. Color codes correspond to the ones used for pleasure ratings.

As illustrated in Figure 3a through d, pleasure over time always followed the same stereotypic time course. The steady-state pleasure during stimulus presentation depends strongly on the final beauty judgment, *F*(3, 373) = 76.66, *p* < 0.001. The greater the felt beauty, the higher the pleasure in general (see Figure 3e). There was no main effect of stimulus duration on pleasure, *F*(3, 373) = 0.68, *p* = 0.565, nor did duration affect differences between beauty categories, *F*(9, 373) = 0.65, *p* = 0.759. As the use of raw data could violate the independence assumption for ANOVAs, we also repeated analyses with averages per participant. We therefore calculated the mean pleasure amplitude per participant for each stimulus duration, stimulus type, and final judgment: There was a main effect of beauty judgment, *F*(3, 55) = 32.84, *p* < 0.001; no main effect of stimulus duration, *F*(3, 55) = 0.36, *p* = 0.783; and no interaction, *F*(9, 55) = 0.75, *p* = 0.665. Thus, even with maximum power, stimulus duration had no effect on pleasure, whereas beauty judgment greatly affected it, even with minimal power.

**Figure 4:**
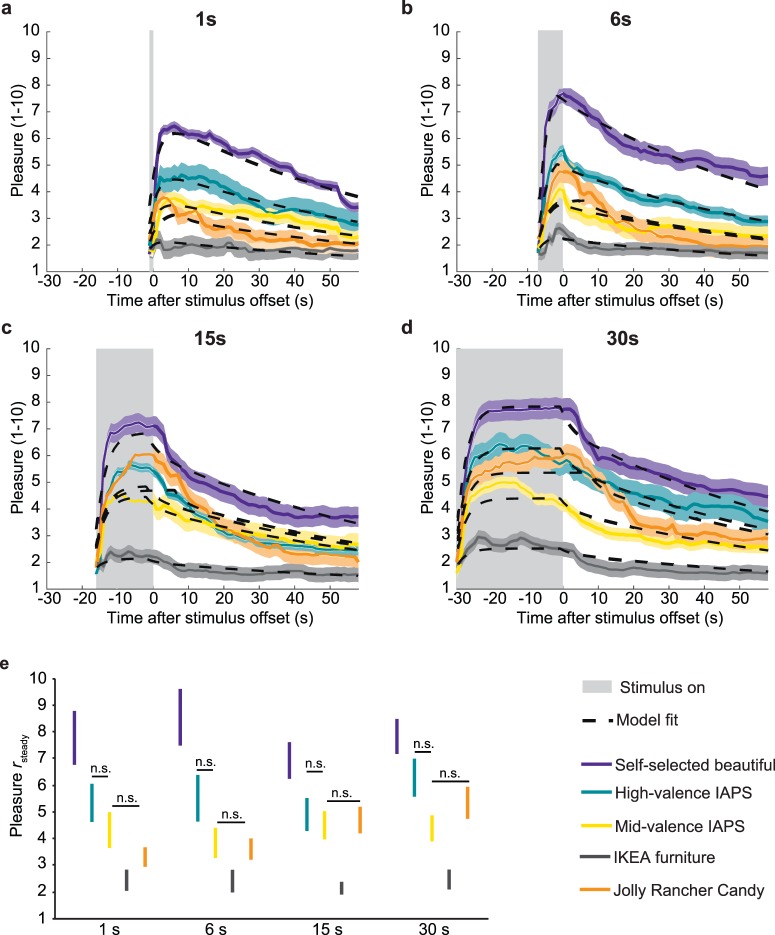
Model fits and r_steady_ for each stimulus type. (a–d) Time course of pleasure rating (M ± SEM) and corresponding model fits for each beauty judgment for each duration (a: 1 s; b: 6 s; c: 15 s; d: 30 s). Model fits were obtained with Equations 1 through 3. Solid lines represent the data, dashed black lines model fits. Shaded areas represent ± SEM. Colors indicate stimulus category: violet = self-selected beautiful; turquoise = high-valence IAPS; yellow = mid-valence IAPS; orange = Jolly Rancher candy; gray = IKEA furniture. The light gray shaded area indicates the interval during which the stimulus was present. (e) M ± SEM for r_steady_ for each stimulus type and duration. Color codes correspond to the ones used for pleasure ratings.

Figure 4 illustrates the results obtained for trials sorted by stimulus type rather than the participants' subjective experience of beauty. Pleasure for the different stimulus types varied substantially, *F*(4, 369) = 39.38, *p* < 0.001. Pleasure from self-selected beautiful images was consistently higher than for all other stimulus types, while pleasure from the neutral IKEA furniture images was always lowest (see Figure 4e). Again, stimulus duration did not affect pleasure in general, *F*(3, 369) = 1.13, *p* = 0.335, or modify the differences between stimulus types, *F*(12, 369) = 0.80, *p* = 0.648. Again, the results held true when averaging our data across participants and calculating mean pleasure for each stimulus duration, stimulus type, and final judgment: Stimulus type affected pleasure ratings, *F*(4, 51) = 3.33, *p* = 0.017, but stimulus duration did not, *F*(3, 51) = 0.26, *p* = 0.851, and neither did the interaction, *F*(12, 51) = 0.26, *p* = 0.992.

As steady-state pleasure values were based on slightly different fits (averaged either for each stimulus type or for each beauty judgment) we have so far reported separate analyses on stimulus and beauty judgment effects. To explore potential three-way interactions, we also ran the full 3 × 3 × 4 (Beauty judgment × Duration × Stimulus type) ANOVA on each set of steady-state pleasure. As before, pleasure changed according to beauty judgment, both *F*(3, 318) ≥ 77.87, both *p* < 0.001, and according to stimulus type, both *F*(4, 318) ≥ 8.72, both *p* < 0.001, with no effect of stimulus duration, both *F*(3, 318) ≤ 0.78, both *p* ≥ 0.503. No interaction reached significance, all *p* ≥ 0.166.

## Discussion

We investigated how the experiences of beauty and pleasure depend on stimulus duration. We find that the feeling of beauty and the amplitude of pleasure are independent of stimulus duration over the range 1–30 s. Moreover, across all durations and stimulus types, the time course of pleasure has a stereotypical shape (Equation 1), sustained for the full stimulus duration.

### Stimulus duration does not affect pleasure

The unfolding of pleasure over time, measured with our touchscreen web app EmotionTracker.com, was well fit by a simple model: Pleasure exponentially reaches a plateau of maximal pleasure and after stimulus offset decays again exponentially. The exponential decay has two components: (weakly) a fast one with the same time constant as the initial increase, and (mostly) a slow one with a longer time constant. Exponential time constants specify time to travel 1 − 1/*e*, or about two thirds of the way. After stimulus onset, pleasure approaches its plateau with a roughly 2–3 s time constant and, after stimulus offset, approaches its asymptotic end state with a time constant of about 70 s. The amplitude of the pleasure plateau (steady-state pleasure) grows with the beauty judgment. The time constants of onset and decay are independent of stimulus duration and beauty judgment. Steady-state pleasure is independent of stimulus duration, over the range 1–30 s. Only beauty judgments weakly increased with increasing stimulus duration. All of these results also held true for the gustatory candy stimulus.

The near absence of duration effects in our study is in line with the “duration neglect” observed by Fredrickson and Kahneman (1993) for film clips with positive emotional content. Over long durations (55–138 s), they found that duration did not affect continuous pleasure ratings during or after viewing film clips. We here show that such duration invariance extends to shorter presentation (1–30 s) of static images. So, it may be that our results reflect a more general conservation of the intensity of affective responses across stimulus durations beyond 1 s. We measured pleasure and beauty and found the same results for both reports. Thus, aesthetic judgments obey at least some of the rules that apply to other emotion-related ratings. Our experiment looked only at the effect of duration. We would encourage further attempts to dissociate aesthetic and emotional ratings. Our visual and gustatory results also parallel the musical finding that people can tell whether they will like a complete piece after hearing just a 750 ms excerpt (Belfi et al., 2016).

### Implications for theory and experimental practice

The stimulus durations in the current study (1–30 s) extend well beyond the under-1-s durations previously used to study effects of processing ease. The typical increase of liking in those studies is about 0.1 points per 100 ms on a 7-point Likert scale. Extrapolating this effect linearly, one would expect a 4-point difference between our 1 and 5 s conditions, whereas we found no effect (see Figures 3a and 4a), refuting a linear extrapolation of the duration effects observed for shorter durations. It may be that maximum processing fluency is reached after 1 s of stimulus presentation. We show here that pleasure ratings do not increase with stimulus durations of 1–30 s.

This opens the question of whether aesthetic judgments could be as fast and intuitive as judgments of facial attractiveness (Willis & Todorov, 2006). In a prior study, we found that the experience of beauty requires cognitive capacities (Brielmann & Pelli, 2017). Our data here are consistent with the necessity of thinking processes for the experience of beauty. In the 1-s condition, pleasure ratings peaked after stimulus offset. Presumably participants were still contemplating the stimulus. Such a prolonged memory-based engagement with the stimulus can reconcile our results with the necessity of thought. We showed that pleasure ratings are conserved across duration of 1–30 s.

Museum visitors spend on average about 30 s in front of a single exhibit (Brieber et al., 2014; Smith & Smith, 2001), which is largely in the 1–30 s range of our results. Such variations in viewing time would be expected to affect only the duration of the pleasure, not its amplitude. Yet, viewing beauty for greatly prolonged durations (e.g., minutes) could potentially be a different and even life-changing experience. It is claimed that viewing for at least 20 min enhances “connection” to a painting (Rosenbloom, 2014). In Greek myth, after looking long at beauty, Pygmalion fell in love and Narcissus died. And long viewing seems to be part of anecdotes about the “Stendhal syndrome,” in which too much beauty makes you sick. Thus, it might be interesting to study the effects of even longer durations, beyond 30 s.

## Conclusion

In conclusion, we here provide a very simple model of the dynamic unfolding of pleasure over time during aesthetic experience. Continuous pleasure ratings obtained with our custom web app EmotionTracker.com can be modeled by exponential transitions, at stimulus onset and offset, between steady states (Equation 1). The steady-state pleasure during the stimulus varies with stimulus type and beauty judgment, while all other parameters are stable across stimulus types, durations, and beauty judgments. The pleasure amplitude is independent of stimulus duration in the tested range, 1–30 s. This parallels the quick judgment of musical preference, where people can tell whether they'll like a complete piece after hearing just a 750-ms excerpt (Belfi et al., 2016). This finding goes against the popular idea that the pleasure of beauty grows with prolonged contemplation, and constrains processing theories in aesthetics.

**Table 1:** Root mean square errors (RMSE) for the model fit to pleasure (0 to 10) either for each duration and beauty judgment (top) or for each duration and stimulus type (bottom). Notes: IAPS = International Affect Picture System.

Condition	Stimulus duration			
	1 s	6 s	15 s	30 s
Beauty judgment				
Definitely not	0.15	0.19	0.26	0.25
Perhaps not	0.15	0.13	0.18	0.22
Perhaps yes	0.24	0.26	0.32	0.35
Definitely yes	0.28	0.45	0.41	0.34
Stimulus				
Self-selected beautiful	0.37	0.49	0.42	0.39
High-valence IAPS	0.22	0.27	0.47	0.34
Mid-valence IAPS	0.22	0.23	0.18	0.33
IKEA furniture	0.15	0.15	0.12	0.23
Jolly Rancher candy	0.25	0.46	0.70	0.47

## Supplementary Material

Supplement 2

## Footnotes

1 Our data (.mat file) is available on GitHub: https://github.com/denispelli/brielman2017duration/
