# Supplementary material for: Beauty at a glance: The feeling of beauty and the amplitude of pleasure are independent of stimulus duration
Source: J Vis. 2017 Dec 11;17(14):9. doi: 10.1167/17.14.9 (PMC6894407; doi:10.1167/17.14.9)
Supplement: Supplement 2 [file jovi-17-12-16_s02.pdf]

Supplementary Material

Beauty at a glance. The feeling of beauty and the amplitude of pleasure are independent of stimulus duration

Aenne A. Brielmann, Lauren Vale, & Denis G. Pelli

Pre-tests of the EmotionTracker.com web app's usability

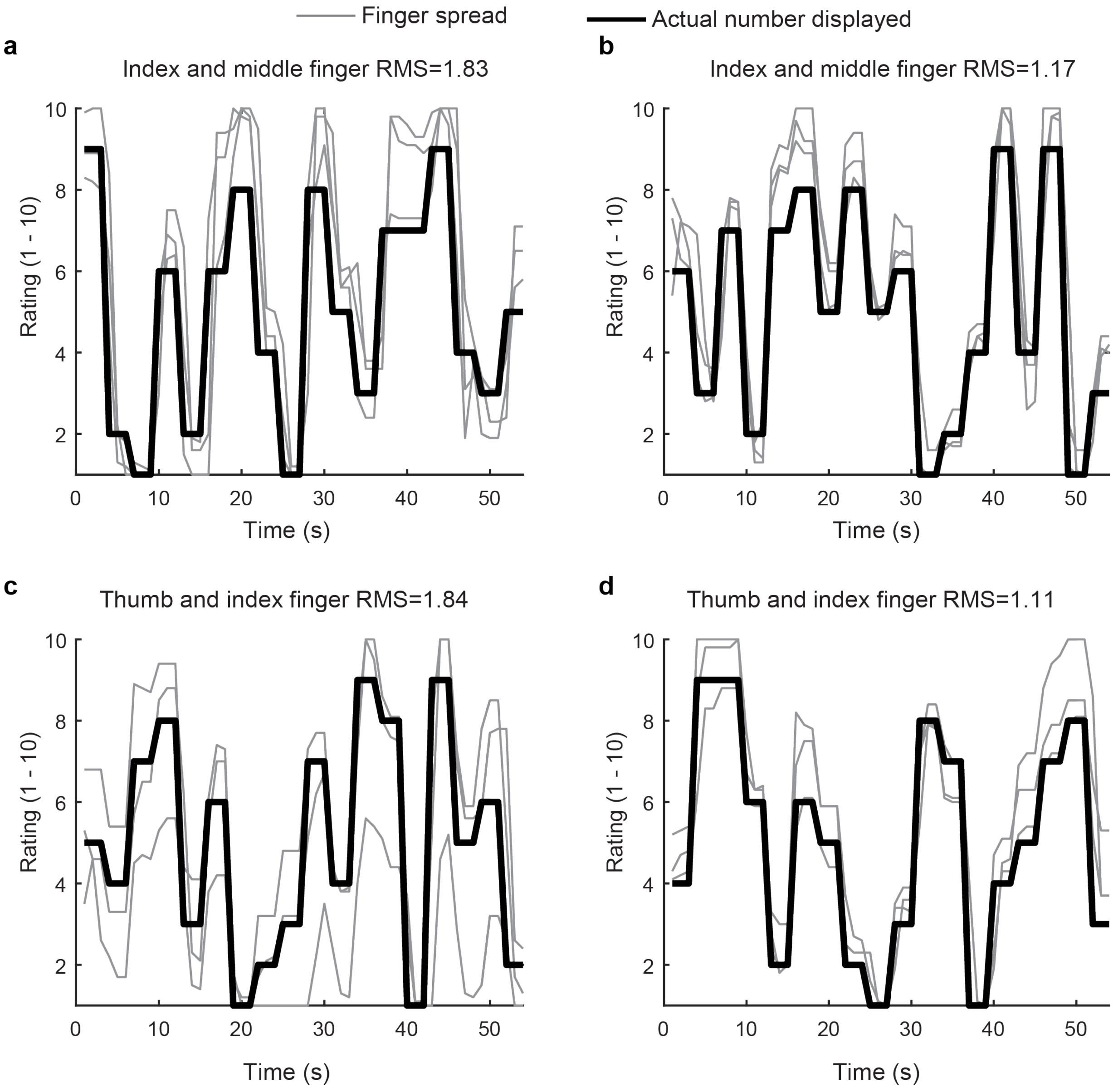

Figure S1. Continuous ratings obtained with the EmotionTracker.com web app (gray) for a sequence of random numbers (black). Each number was presented for three seconds. Each gray line refers to data of one of the three observers. Data is displayed for ratings with index and middle finger (a, b) as well as for ratings with thumb and index finger (c, d).

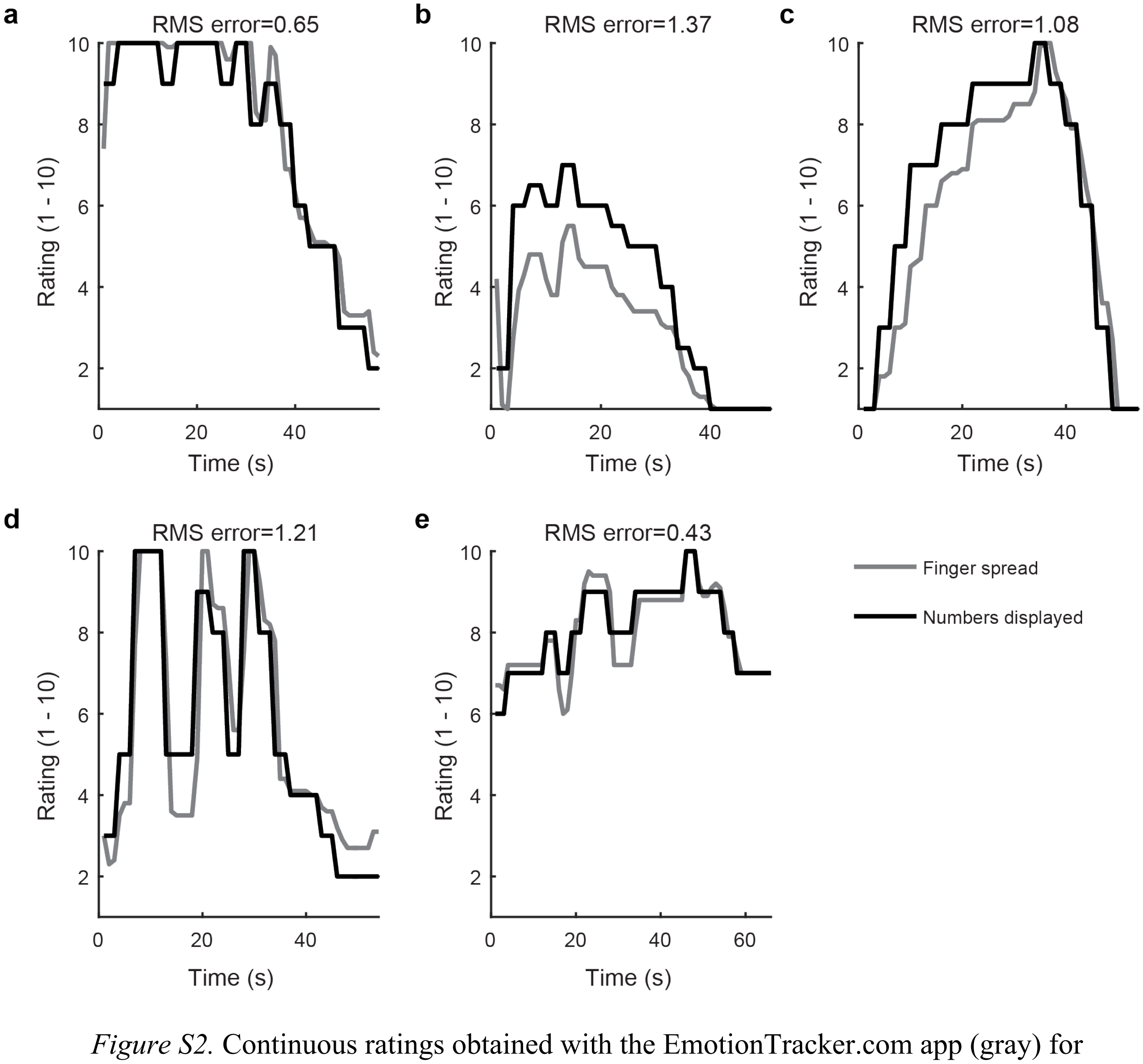

Figure S2. Continuous ratings obtained with the EmotionTracker.com app (gray) for rounded numbers from previous trials (black). Each gray line refers to data of one observer who is one of the authors. All ratings were obtained with thumb and index finger.

Residuals of model fit over time

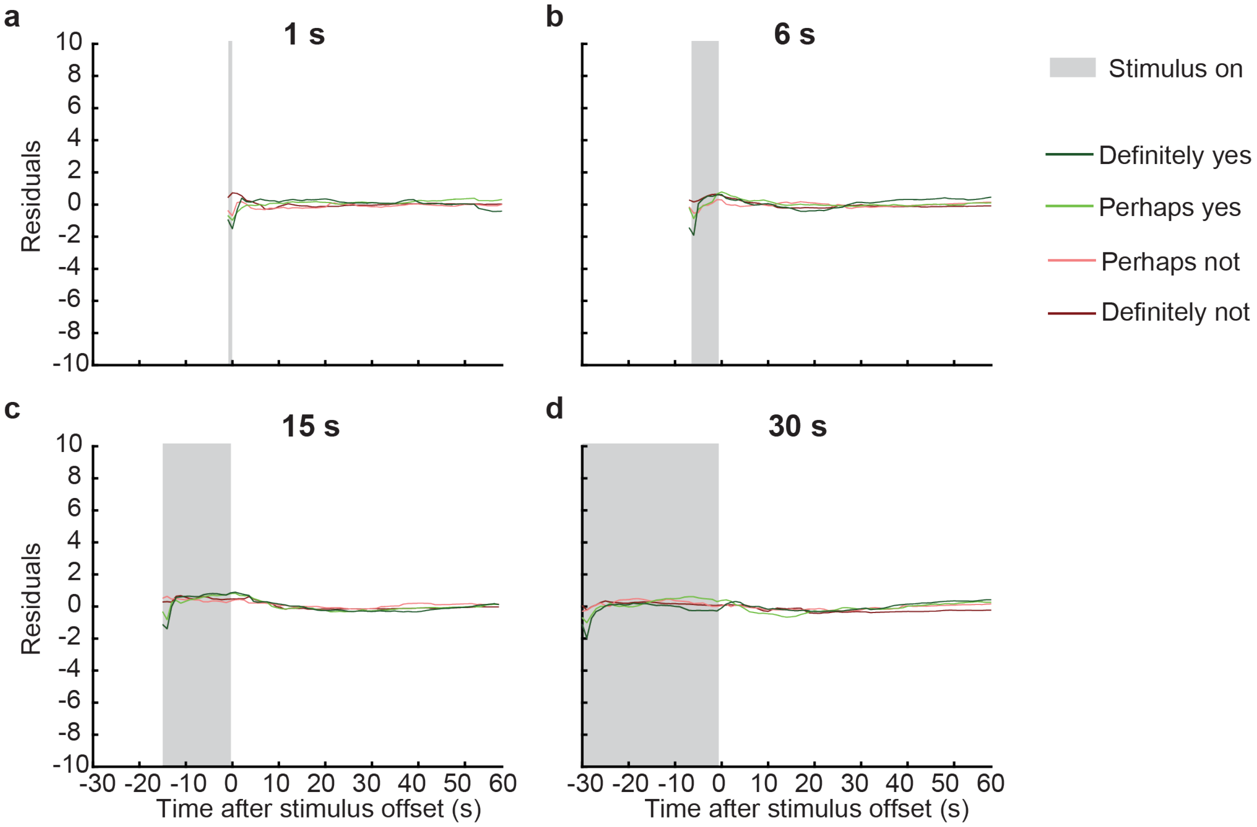

Figure S3. Residuals of model fit for averages per beauty judgment and stimulus duration (a: 1s; b: 6s; c: 15s; d: 30s). Colors indicate final beauty judgments: dark red = "definitely not"; light red = "perhaps not"; light green = "perhaps yes"; dark green = "definitely yes". The gray shaded area indicates the interval during which the stimulus was present.

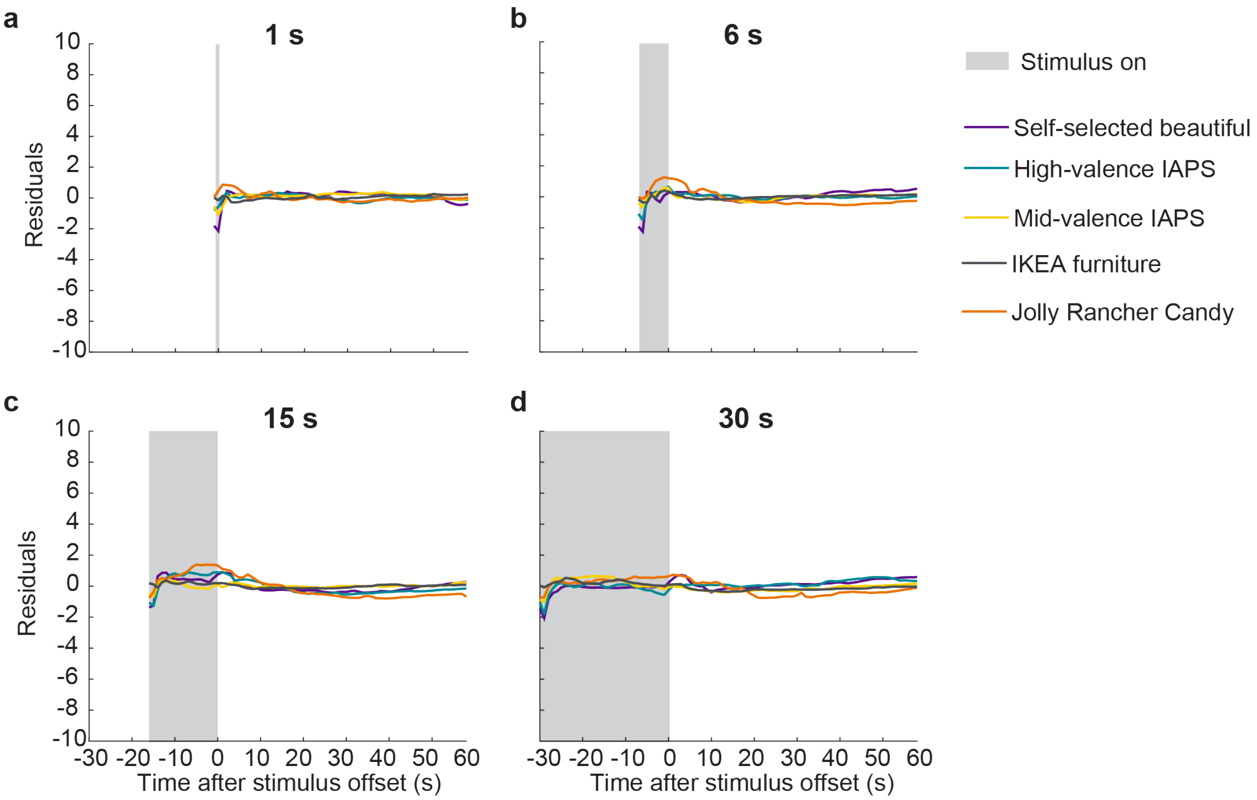

Figure S4. Residuals of model fit for averages per stimulus type and stimulus duration (a: 1s; b: 6s; c: 15s; d: 30s). Colors indicate stimulus type: violet = self-selected beautiful; turquoise = high-valence IAPS; yellow = mid-valence IAPS; orange = Jolly Rancher candy; gray = IKEA furniture. The light gray shaded area indicates the interval during which the stimulus was present.

Average pleasure over time and model fit aligned by stimulus onset

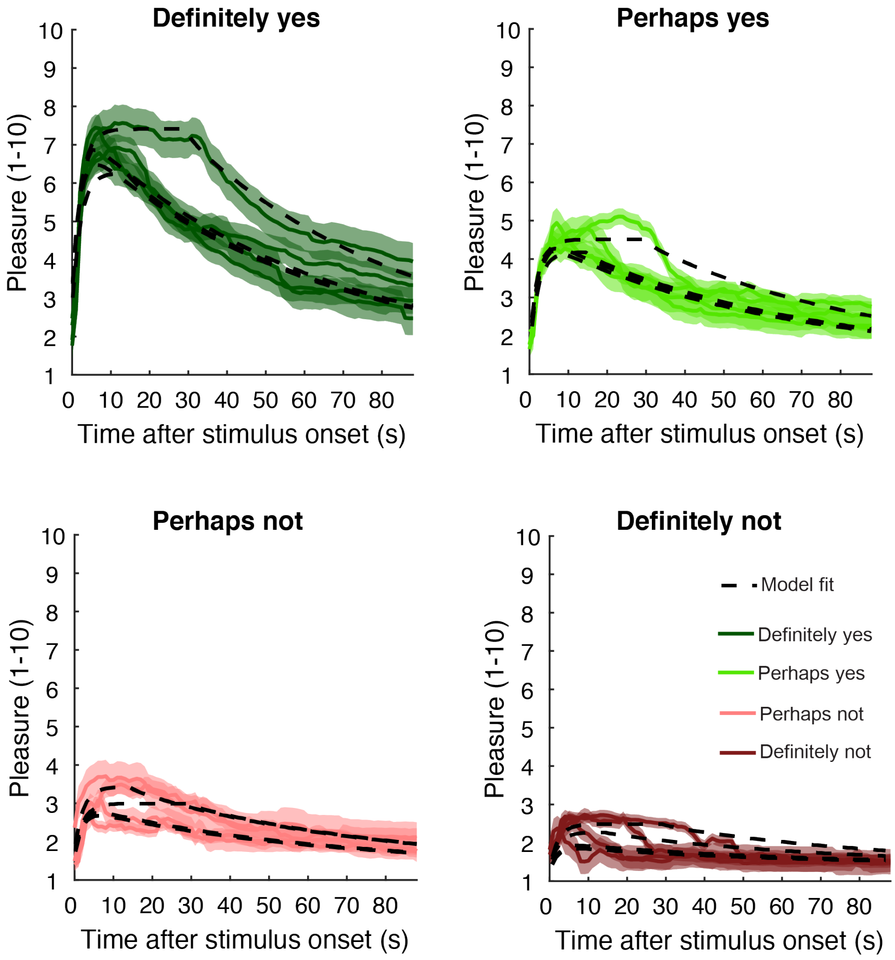

Figure S5. Model fit and average continuous pleasure ratings aligned by stimulus onset per beauty judgment and duration. Model fits were obtained with equations (1-3) displayed in the main article. Solid lines represent the data, dashed black lines model fits. Shaded areas represent ±1 SEM. Colors indicate final beauty judgments: dark red = "definitely not"; light red = "perhaps not"; light green = "perhaps yes"; dark green = "definitely yes".

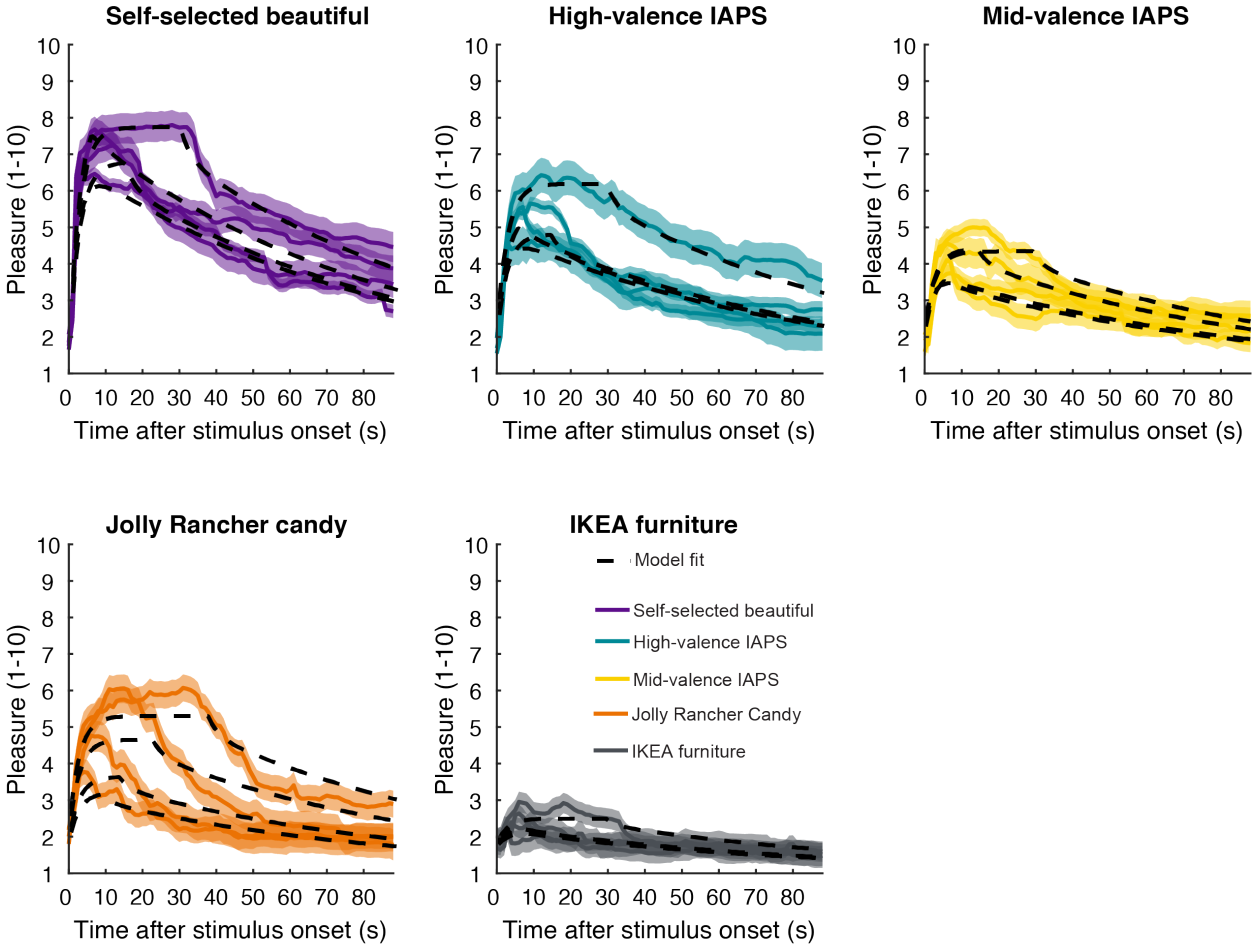

Figure S6. Model fit and average continuous pleasure ratings aligned by stimulus onset per stimulus type and duration. Model fits were obtained with equations (1-3) displayed in the main article. Solid lines represent the data, dashed black lines model fits. Shaded areas represent ±1 SEM. Colors indicate stimulus category: violet = self-selected beautiful; turquoise = high-valence IAPS; yellow = mid-valence IAPS; orange = Jolly Rancher candy; gray = IKEA furniture.
